# In Vivo Behavior of Biomimetic Nanoparticles: Strategies for Clearance Avoidance, Targeting, and Functional Delivery

**DOI:** 10.3390/molecules30224487

**Published:** 2025-11-20

**Authors:** Polina Lazareva, Vladimir Chulanov, Dmitry Kostyushev, Maxim Abakumov

**Affiliations:** 1 Department of Medical Nanobiotechnology, N.I. Pirogov Russian National Research Medical University, Moscow 117997, Russia; polizara604@gmail.com (P.L.); abakumov1988@gmail.com (M.A.); 2 Laboratory of Experimental Therapy of Infectious Diseases, Martsinovsky Institute of Medical Parasitology, Tropical and Vector-Borne Diseases, Sechenov University, Moscow 119435, Russia; vladimir@chulanov.ru; 3 Center for Precision Genetic Technologies for Medicine, Engelhardt Institute of Molecular Biology, Russian Academy of Sciences, Moscow 119991, Russia; 4 Department of Infectious Diseases, Sechenov University, Moscow 119435, Russia; 5 Laboratory of Genetic Technologies, Martsinovsky Institute of Medical Parasitology, Tropical and Vector-Borne Diseases, First Moscow State Medical University (Sechenov University), Moscow 119991, Russia; 6 Faculty of Bioengineering and Bioinformatics, Lomonosov Moscow State University, Moscow 119192, Russia; 7 Laboratory of Biomedical Nanomaterials, National University of Science and Technology (MISIS), Moscow 119049, Russia

**Keywords:** targeted drug delivery, nanomedicine, cancer, blood–brain barrier, arthritis

## Abstract

Biomimetic cell membrane-coated nanoparticles (BMCNPs) are an attractive drug delivery platform that combines the advantages of an inorganic core with the biological functionality of a natural cell membrane. This hybrid design merges the versatility of engineered nanomaterials with the complexity and specificity of biological systems, enabling prolonged circulation, immune evasion, enhanced tissue targeting, and improved therapeutic efficacy. In this review, we explore the in vivo behavior of BMCNPs, focusing on their interactions with biological barriers, including evasion of mononuclear phagocyte system clearance, biodistribution patterns, and circulation kinetics. We also examine how membrane source and surface properties influence targeting efficiency and delivery outcomes, while highlighting key considerations and emerging strategies to optimize therapeutic performance and translational potential.

## 1. Introduction

In recent decades, nanoparticles (NPs) have gained significant attention in the development of novel therapeutic and diagnostic platforms, particularly in drug delivery, molecular imaging, and cancer therapy. This is largely due to their high surface area-to-volume ratio, surface functionalization potential, and tunable physicochemical properties. However, conventionally synthesized NPs often face major limitations, including rapid systemic clearance, limited tissue penetration, immunogenicity, and insufficient biocompatibility [1].

**Figure 1:**
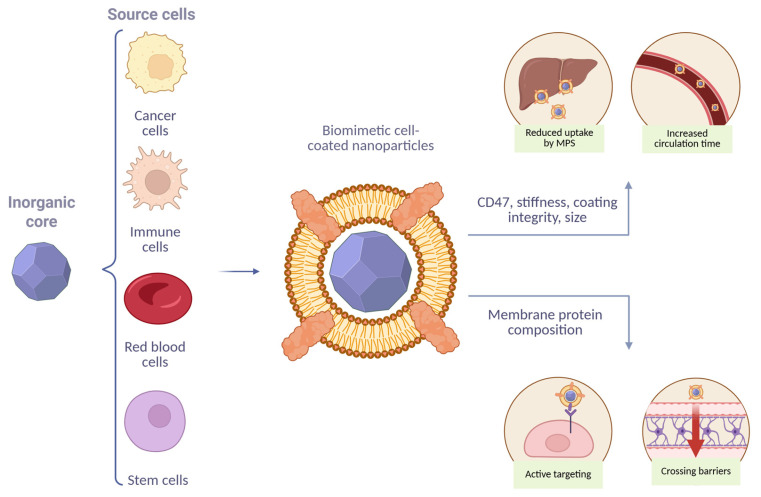
Schematic illustration of BMCNPs and their unique characteristics.

To overcome these challenges, a new class of bioinspired systems—biomimetic NPs—has been developed, designed to mimic the biological properties of natural cells or cellular components (Figure 1) [2]. This approach enables the exploitation of evolutionarily optimized biological mechanisms to achieve improved biocompatibility, prolonged systemic circulation, immune evasion, and targeted delivery [3].

Depending on their fabrication strategies, biomimetic NPs can generally be categorized into three main types: (1) biomimetic cell membrane-coated nanoparticles (BMCNPs), (2) artificial cell-derived vesicles, and (3) fully synthetic vesicles [4]. BMCNPs, one of the most successful approaches, involves coating a synthetic NP core (e.g., poly(lactic-co-glycolic acid) (PLGA), gold, or silica) with natural cell membranes derived from red blood cells (RBCs), platelets, tumor cells, immune cells, or stem cells. This membrane camouflage allows the particles to mimic “self” signatures, thereby avoiding macrophage-mediated phagocytosis and, as a result, prolonging circulation time while reducing nonspecific accumulation in off-target organs. Moreover, it enables active targeting through specific interactions with diseased tissues and provides cross-barrier transport. In addition, the membranes of source cells can transfer their intrinsic therapeutic properties to BMCNPs [5,6,7,8,9].

## 2. Fabrication Methods

When discussing the behavior of BMCNPs in biological systems, a key aspect is the analysis of their fabrication methods. The method of membrane preparation can affect the integrity of their composition [4]. The degree of cell membrane coverage on the inorganic core influences cellular uptake [10].

The production of BMCNPs can be divided into several main stages:

Cell lysis;

Membrane extraction;

Membrane modification;

Inorganic particles coating (Biomimetic Nanoparticles for Basic Drug Delivery).

### 2.1. Cell Lysis

The main widely used cell lysis methods include the following:

Hypotonic lysis

This method is based on cell swelling and rupture under low osmotic pressure. This is a simple and effective method, but it has low efficiency and is most commonly used for extracting erythrocyte membranes [11].

Freeze–thaw

Membrane extraction relies on freeze–thaw cycles. The advantages include simplicity, but temperature changes can affect the activity of surface proteins [11,12].

Nitrogen cavitation

Nitrogen cavitation requires a specialized chamber where nitrogen bubbles are formed. The mechanism involves two stages: under pressure, nitrogen diffuses into cells, and upon pressure reduction, it forms bubbles directly in the cellular space. These bubbles create mechanical stress, leading to cell destruction. Despite its effectiveness, this method is rarely used in lysis practice due to its long duration, the need for expensive equipment, and the risk of altering cell membrane morphology [4].

Sonification

This method involves cell lysis via ultrasonic frequencies ranging from 20 to 50 kHz. It is an effective method, but ultrasound exposure generates ROS and releases significant heat, leading to protein denaturation. This can be mitigated using ice buffers [13]. However, this may limit the scalability of this lysis method [11].

Homogenization

Homogenization (micronization) reduces particles to microscopic sizes to improve emulsions and dispersions. The Dounce homogenizer—a glass tube with two pestles (loose and tight)—is designed for gentle disruption of eukaryotic cells and organelle isolation. It is preferred in enzyme studies (no overheating occurs). The procedure involves tissue grinding, addition of lysis buffer, and several passes with a loose pestle, followed by a tight pestle (for cell disruption). Hypotonic buffers enhance lysis by causing cytoplasmic swelling and facilitating membrane rupture [13].

### 2.2. Membrane Extraction

At the second stage, membrane isolation is performed using the following:

Density gradient ultracentrifugation (DGU);

Standard ultracentrifugation (UC);

Medium-speed centrifugation.

DGU provides high membrane purity but requires considerable time and specialized equipment. UC and medium-speed centrifugation are faster and simpler but yield lower purity and specificity [4].

### 2.3. Inorganic Particle Coating

After cell lysis and membrane isolation, the membrane is used to coat the inorganic core. Several methods exist for coating inorganic cores with cell membranes. Key approaches include the following:

Co-extrusion

A mixture of inorganic core and cell membranes is repeatedly extruded through porous membranes of varying sizes, followed by ultrasound treatment. Mechanical force allows the inorganic core to pass through the lipid bilayer, inducing membrane and core fusion [14]. NPs have been coated via co-extrusion using RBC, platelets, cancer cells, stem cells, and immune cells [15]. Although co-extrusion ensures controlled BMCNP size and desired orientation (“right side out”), it has significant drawbacks, including intense mechanical stress and high material waste due to deposition on porous membranes [4].

Sonication

Inorganic cores and cell membranes are exposed to ultrasound, after which the membrane reassembles around the nanoparticle, forming a core–shell structure [13]. While sonication requires less time and material than co-extrusion, it results in a poorer coating, with low “right side out” orientation, layer heterogeneity, and potential NP damage. Combining co-extrusion and sonication using microfluidic devices may address these limitations [4,13]. However, this method is not standardized for industrial use and retains all drawbacks of ultrasound processing [4].

A notable advantage of microfluidics-based fusion is the ability to combine synthetic NP fabrication with the subsequent fusion of synthetic liposome-based cores with cell membrane vesicles, yielding hybrid liposomes [16]. Hybrid liposome production eliminates partial membrane coverage of the inorganic core, a common issue with extrusion and sonication [17].

Microfluidic electroporation

Microfluidic electroporation involves introducing a mixture of nanoparticles and plasma membrane vesicles into a microfluidic device, followed by electric field treatment. The current creates temporary pores in membranes, enabling them to envelop nanoparticles. This technology offers high productivity, rapid synthesis, and scalability potential. However, challenges remain: lack of unified standards, batch-to-batch quality variation, and nanoparticle aggregation risk [4]

In 2021, Hu et al. [18] proposed a BMCNP synthesis method addressing scalability. Flash NanoComplexation (FNC) resolves the issue of positively charged NPs aggregating with negatively charged cell membranes. After ultrasound treatment of the membrane, its fragments and NP solution are fed into separate mixer inputs; turbulent mixing then creates uniform coating. Results show that FNC yields BMCNPs with better stability and more predictable size compared to traditional ultrasound processing.

## 3. Pharmacokinetics Models

Two primary approaches are employed for analysis of NP pharmacokinetic data: noncompartmental analysis, which evaluates exposure based on observed parameters without assuming elimination mechanisms, and compartmental analysis, capable of predicting the concentration–time curve using kinetic models [19].

Researchers predominantly utilize one- or two-compartment models [4]. The fundamental assumption underlying the one-compartment model is that an organism is a single compartment in which equilibrium is quickly achieved after systemic introduction [19]. The two-compartment model describes drug distribution in terms of a central compartment (e.g., blood and highly perfused organs) and a peripheral compartment (e.g., tissues with slower drug exchange) [20].

In general, BMCNPs exhibit similar pharmacokinetic behavior compared to uncoated nanocarriers, often following either one-compartment or two-compartment models [4]. However, coating nanoparticles with cell membranes significantly prolongs their half-life in blood across a wide range of nanomaterials for both one- and two-compartment models (Table 1).

In studies employing the one-compartment model, a statistically significant increase in t_1/2_ (half-time) was observed after coating with cell membrane [21,32,33]. In the case of the two-compartment model, distribution time remained largely unchanged, while elimination half-life increased significantly following coating [10,23,25].

It should be noted that after nanoparticle coating, peculiarities of their pharmacokinetics are preserved. For example, coating increased the t_1/2_ of spherical particles by 2.83-fold, prolate ellipsoidal particles by approximately 2.2-fold, and oblate ellipsoidal particles by approximately 1.5-fold. After coating, despite an overall increase in accumulation in the spleen and a decrease in accumulation in the kidneys, differences between the profiles of biodistribution among different organs were preserved [21].

**Figure 2:**
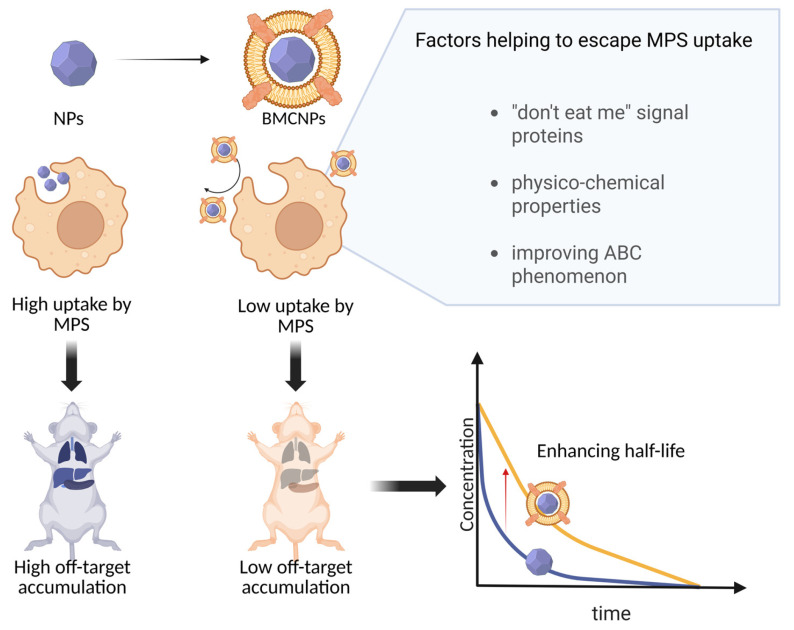
Interaction MPS with NPs after cell coating.

The primary mechanism responsible for the increased circulation time following coating is the delay in recognition and clearance by the mononuclear phagocyte system (MPS), which extends both the distribution and elimination phases (Figure 2) [34].

As noted in recent literature, artificial intelligence/machine learning algorithms are increasingly employed to predict NP behavior in complex biological systems [35]. Notably, deep neural networks have demonstrated strong predictive performance, achieving an R^2^ of 0.92, in forecasting delivery efficiency. Key parameters identified in this context include the zeta potential and the NP material [36]. In contrast, traditional machine learning methods have shown limited predictive capability in similar tasks [37].

When applying artificial intelligence techniques to predict the in vivo behavior of BMCNPs, a large, well-annotated dataset is essential. For instance, researchers modeling classical NPs often rely on physiologically based pharmacokinetics and leverage the Nano Tumor database for training [37,38,39]. However, a significant limitation remains: the current database includes very few entries for BMCNPs (<10). This underscores a broader challenge—the scarcity of well-annotated data for training artificial intelligence models specifically tailored to BMCNPs.

## 4. Factors Affecting MPS Uptake and Its Role in BMCNP Clearance

Upon systemic administration, NPs can be eliminated from the body via three primary routes: renal excretion, hepatobiliary clearance, and elimination through the MPS. The renal pathway is predominantly observed for particles smaller than 5–6 nm in diameter and is not applicable for most types of BMCNPs.

Circulating nanoparticles with sizes below the diameter of liver sinusoidal fenestrations (up to 150–200 nm) or those that evade capture by hepatic Kupffer cells can penetrate into the space of Disse. There, they interact directly with hepatocytes, initiating hepatobiliary elimination. During hepatobiliary clearance, hepatocytes clear foreign nanoparticles through endocytosis, followed by enzymatic degradation. The degradation products are then excreted into bile via the biliary system and ultimately eliminated through feces [40].

The MPS represents one of the first biological barriers encountered by NPs following corona formation and plays a crucial role in NP clearance from the bloodstream [41,42,43]. MPS-mediated capture significantly impacts both the circulation time and the overall therapeutic efficacy of nanomedicines. The cells involved in MPS-mediated elimination primarily consist of bone marrow progenitor cells, circulating blood monocytes, and tissue-resident macrophages predominantly located in the liver (Kupffer cells) and spleen [44].

Due to rapid uptake by MPS cells, the plasma half-life of many NPs is reduced to less than several minutes, and in some cases, less than 1% of the administered dose reaches the intended target tissue [41]. After intravenous administration, synthetic NPs rapidly acquire a protein corona composed of various serum proteins [45]. These adsorbed proteins mediate interactions between nanoparticles and cells through receptor-mediated endocytosis.

Besides erythrocytes, macrophage membranes [29,46,47], stem cell membranes [48,49], and cancer cell membranes [10,50] have been used for camouflage, all reducing phagocytosis and thus nonspecific toxicity. For instance, Cao et al. (2023) demonstrated that macrophage membrane-coated NPs (MM-coated NPs) have a significantly lower level of circulating ICG+ monocytes (indicative of phagocytosis) in vivo than uncoated NPs [46]. The half-life of MM-coated NPs (9.82 h) was markedly longer than that of uncoated NPs (t_1/2_ = 5.43 h) [29]. Similarly, stem cell membranes not only decreased uptake by immune cells but also enhanced homing to tumor tissues due to the presence of natural adhesion molecules [49]. Stem cell-coated particles (SM-coated NPs) showed 4.5- and 2.25-fold increases in blood retention at 12 h compared with uncoated particles and free drug, respectively [48], although they did not reduce hepatic or splenic uptake as effectively as RBC-coated NPs. Another study [51] reported that stem cell coating decreased complement protein binding compared to uncoated particles, though liver uptake remained unchanged, while splenic accumulation was reduced.

### 4.1. Functional Significance of Membrane Molecular Composition

To overcome this barrier, researchers are actively developing strategies to disguise NPs from MPS cells. One of the most studied approaches is the functionalization of NP surfaces with CD47 protein [52,53]. This membrane protein, known as the “don’t eat me” signal, is presented on the surface of various hematopoietic cells, including erythrocytes, platelets, monocytes, and even cancer cell lines [54]. CD47 interacts with SIRPɑ protein and inhibits macrophage phagocytosis, thereby prolonging systemic circulation and reducing immune clearance of cells [55]. Coating NPs with synthetic CD47 analogs has also been shown to increase circulation time by lowering MPS uptake [56]. Moreover, CD47 was identified as the key molecule responsible for the immune evasion of RBC-coated NPs from phagocytosis, making their membranes one of the most promising for fabrication of BMCNPs [57].

The importance of CD47 density on membranes is further supported by experimental evidence. Jiang et al. [26] compared hybrid particles coated with varying ratios of erythrocyte membranes (high in CD47) and MCF-7 cancer cell membranes (CD47-negative). A higher proportion of erythrocyte membranes correlated with prolonged circulation and reduced liver and spleen accumulation. Conversely, the use of aging erythrocyte membranes, which have lower CD47 expression, resulted in increased hepatic accumulation, although t_1/2_ = 69.315 h was not different from uncoated particles [32]. It is important to note that aging erythrocytes undergo changes not only in CD47 levels but also in other surface proteins. Interestingly, coating NPs exclusively with MCF-7 membranes also prolonged half-life compared to uncoated particles (5.1 h vs. 4.0 h), although the effect was less pronounced than RBC membrane coating (11.2 h).

As with RBC membranes, CD47 plays a critical role in reducing phagocytosis of cancer cell-coated NPs (CM-coated NPs). Blocking CD47 and CD44 on 4T1 CM-coated NPs increased phagocytosis in vitro to levels similar to protein-free controls, whereas unblocked particles showed reduced uptake [50]. Interestingly, in this case, the presence or absence of membrane proteins did not influence liver accumulation but significantly altered splenic accumulation, which was attributed to targeting CD4+ T cells rather than macrophage uptake. However, in another study [58], an increasing of production of CD47 and integrin α4/β1 on macrophage membranes reduced the accumulation in the liver compared to particles coated with membranes exhibiting normal levels of these proteins and uncoated controls.

A comparison between RBC- and platelet-coated systems revealed that although both carry CD47, RBC-coated NPs circulated longer (42.4 h vs. 38.3 h), while hybrid coatings combining RBC and platelet membranes further extended circulation to 51.8 h. All three formulations, however, displayed similar biodistribution profiles across organs [59].

It should be emphasized that CD47 also inhibits the complement system, a major trigger of opsonization. This process leads to rapid plasma clearance of nanocarriers, significantly reducing their half-life and limiting accumulation in target tissues such as tumors or the central nervous system (CNS) [60]. The significance of this mechanism was confirmed by in vivo studies of protein coronas formed on liposomes versus RBC-coated NPs [27]. The results demonstrated significant differences in composition: increased levels of CD47 and complement proteins, alongside reduced myosin-binding proteins, correlated with enhanced resistance to phagocytosis. Notably, the half-life of liposomes was more than twofold shorter. In vitro studies from 2017 [61] and 2019 [62] also showed that the corona of RBC-coated NPs lacked complement proteins, unlike uncoated NPs.

Besides proteins, the lipid composition of membrane is also an important factor that should be taken into consideration. For example, phosphatidylserine (PS) is a lipid whose externalization serves as a phagocytic signal. During RBC-coated NPs preparation, PS exposure may occur; however, cholesterol enrichment of membranes mitigates this effect. Cholesterol-enriched particles exhibited reduced hepatic accumulation 4 h post-injection (7.51% vs. 17.17%) and 1.8-fold higher blood retention at 24 h compared with non-enriched particles [63]. Therefore, RBC and other cell membranes contain a wide repertoire of proteins and lipids that reduce MPS uptake.

However, other factors might strongly affect NP uptake by MPS. The extent of membrane coating also critically determines pharmacokinetics. Although full coating should theoretically transfer all donor-cell properties, in practice, common methods such as sonication and extrusion result in only 1.8–6.5% of particles being fully coated [17]. For CM-coated NPs, incomplete coverage increases macrophage uptake in vitro and shortens in vivo half-life (18.5 h vs. 23.6 h) [10]. Another challenge is post-administration membrane degradation. For example, Ma et al. [64] showed that embedding antioxidant nanozymes into RBC-coated NPs extended circulation time, whereas non-antioxidant cores triggered faster immune attack and elevated IgM/IgG levels.

### 4.2. Roles of Physicochemical Parameters

It is also worth noting that although cell camouflage itself reduces nanoparticle clearance, additional physicochemical parameters influence in vivo behavior. Speaking generally about nanoparticles, their elasticity affects their pharmacokinetic properties [65]. Softer (with a low Young’s modulus) NPs avoid macrophage phagocytosis better than harder ones [66]. This may explain why soft NPs have a longer blood retention time and an increased half-life [67,68,69].

After membrane coating, the stiffness properties of the core are transferred to BMCNPs. Similar to uncoated NPs, softer BMCNPs exhibit a longer circulating half-life and reduced uptake by macrophage-like cells compared to harder NPs. For example, coating hard BMCNPs (Young’s modulus 2.3 GPa) with MCs did not reduce uptake by macrophage-like cells in vitro, unlike soft BMCNPs (44 MPa), which avoided phagocytosis significantly better than their uncoated core NPs [70].

In another study, Yuan et al. showed that softer BMCNPs (11 MPa) had the longest circulating half-life of 20.4 h compared to 13 h (for 95 MPa NPs) and 11.5 h (for 173 MPa) [33]. Interestingly, in a different study [25], RBC-coated NPs with different Young’s moduli (<3 MPa, ~10 MPa, and ~25 MPa) demonstrated that medium-stiffness nanoparticles showed the longest half-life (51.1 h), which was 1.4- and 1.6-fold longer than the softest and hardest BMCNPs. Moreover, medium-stiffness NPs exhibited significantly lower fluorescence intensity and less colocalization with macrophages in the liver than other treatment groups [25]. This suggests there is an optimal stiffness for improved MPS avoidance and circulation time, apparently around 10 MPa.

Additionally, core stiffness affects the amount of membrane proteins transferred to BMCNPs after coating. For example, softer BMCNPs (44 MPa) coated with mesenchymal stem cells formed a membrane coating with a higher protein content, resulting in a higher surface concentration of membrane proteins such as CD90 and CXCR4 [70] than harder NPs (2.3 GPa).

For RBC-coated NPs, reducing the size from 200 nm to 80 nm is associated with prolonged half-life and elimination. Although particle size did not affect macrophage uptake activity, reducing the size of rigid biomimetic nanoparticles can improve nanoparticle passage through liver sinusoids, decrease the number of captured nanoparticles, and thus prolong the circulation time of biomimetic nanoparticles [3].

### 4.3. ABC Phenomenon

Another significant advantage of BMCNPs is their ability to evade the accelerated blood clearance (ABC) phenomenon, which is commonly observed with PEGylated (polyethylene glycol) nanomaterials. ABC typically results in enhanced hepatic and splenic accumulation and faster systemic clearance upon repeated administration due to anti-PEG antibody production and opsonization [71]. In contrast, BMCNPs, demonstrate negligible induction of immune memory and maintain a consistent biodistribution profile over multiple doses. For instance, Zhang et al. (2015) reported that magnetic RBC-NPs did not accumulate in liver or spleen upon repeated administration, whereas PEG-NPs displayed strong uptake by macrophage-rich organs [72]. Coating PEGylated gold nanoparticles with RBC membranes eliminated the ABC phenomenon and prevented anti-PEG antibody production [28]. Similar immune-evading properties were observed for CM-coated NPs, which also resisted ABC-induced clearance upon multiple injections [73]. As mentioned above, membrane integrity remains a key determinant of pharmacokinetics; degradation by reactive oxygen species (ROS), for instance, can enhance hepatic uptake and reduce blood retention, whereas ROS-resistant cores maintain longer circulation [64].

### 4.4. Determination of QTTP

To summarize the key findings discussed above, cell membrane coating generally enhances the t_1_/_2_ of NPs, as demonstrated in Table 1. Notably, different membrane types exhibit varying degrees of circulation time prolongation. Specifically, RBC membrane coating increases particle circulation time more significantly compared to cancer cell membrane coating. This observation is further supported by data on hybrid nanoparticles composed of different membrane ratios, which display variable circulation times. For instance, melanin NPs coated exclusively with RBC membranes showed a 2.8-fold increase in t_1/2_. When incorporating increasing proportions of cancer cell membranes (33%, 50%, 66%, and 100%), the t_1/2_ increases were 2.73-fold, 2.68-fold, 2.1-fold, and 1.8-fold, respectively [26]. Furthermore, as detailed in Section 4, several critical material attributes (CMAs) significantly influence nanoparticle performance, including the following:

CD47 amount;

Coating integrity;

Particle size;

Stiffness properties.

Importantly, increasing BMCNP size from 80 nm to 200 nm correlates with enhanced NP accumulation in the liver, with minimal impact on splenic and kidney accumulation. Additionally, BMCNPs smaller than 100 nm demonstrated prolonged circulation times, as shown in Table 1.

As discussed in Section 4.2, an optimal stiffness of approximately 10 MPa for BMCNPs has been identified. This stiffness value correlates with the following:

Maximum circulation time;

Improved tumor accumulation;

Consequently, an enhanced therapeutic index.

Based on all of the above, we have determined the following Quality Target Product Profile (QTPP) (Table 2).

## 5. Targeted Delivery

### 5.1. Targeting by Initial Membrane Characteristics

**Figure 3:**
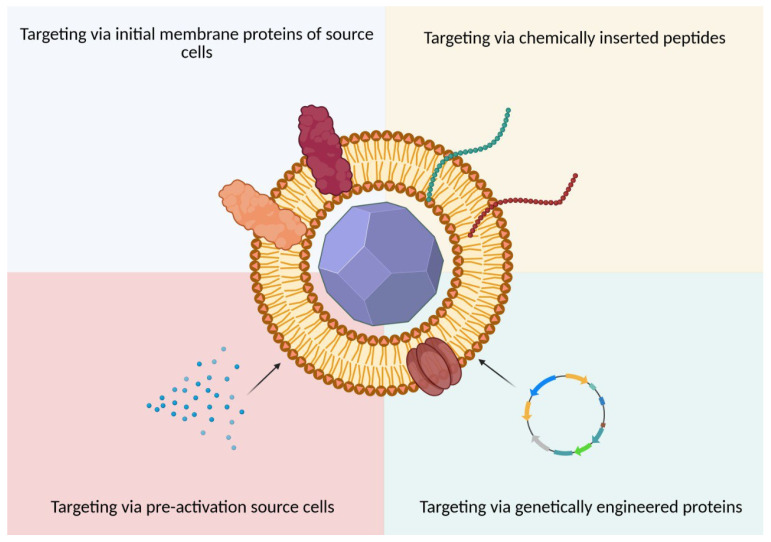
Targeting of BMCNPs.

As shown in the previous section, BMCNPs can effectively prolong blood circulation time by reducing uptake by MPS. However, for efficient therapy or diagnostics, BMCNPs must be delivered to the disease region in the body. Two main mechanisms providing such delivery are namely the enhanced permeability and retention (EPR) effect, also known as passive targeting, and active targeting, also known as targeted delivery. The unnormal vasculature at pathological conditions, such as solid tumors and inflamed tissues, facilitates the passive targeting and accumulation of NPs. However, translation of various nanomedicines from preclinical to clinical settings has revealed that the EPR effect has limited clinical significance [3], such as heterogeneous presentation of EPR in different tumors, lack of cellular specificity, and low vascular density [75]. For this reason, the using of active targeting in combination with passive targeting can solve these problems, increasing cellular specificity (Figure 3).

For active targeting, specific targets are required that distinguish pathological tissue from healthy. Regarding diverse diseases such as cancer, atherosclerosis, pulmonary diseases, rheumatoid arthritis, and others, chronic inflammation plays a key role in their pathogenesis [76]. Moreover, at the early stages of these diseases, vascular inflammation occurs, which is characterized by endothelial activation and the expression of various factors, such as vascular cell adhesion molecule 1 (VCAM-1), intercellular adhesion molecule 1 (ICAM-1) [77], CD44 [78], and selectins [79]. In addition, these factors promote angiogenesis [80,81,82], which correlates with increased collagen IV [83] and other markers.

The expression of these molecules promotes the recruitment and adhesion of different cells [84]. Specifically, VCAM-1 and ICAM-1 bind to integrins on leucocyte surfaces [85,86]. Selectins of inflamed endothelium interact with P-selectin glycoprotein ligand 1 on leucocytes [84]. In addition, P-selectin expressed on platelet surfaces binds to CD44 [87]. Other platelet-specific receptors for collagen and laminin glycoprotein VI, by binding to collagen IV, induce platelet activation under inflamed conditions [88].

Targeting of source cells to vascular inflammation via membrane proteins is transferred to BMCNPs. Thus, MM-coated NPs accumulate at the tumor site [89,90,91,92]. Importantly, increased drug delivery for macrophages is paired with elevating inflammation within the tumor microenvironment (TME), for example, during photothermal therapy [93]. Consistent with this, MM-coated NPs demonstrated 30% for 18 h compared to ~10% for uncoated NPs under pneumonia conditions [94].

Moreover, neutrophil camouflage provides strong tumor targeting and enhances therapy [95]. In addition, neutrophils exhibit excellent targeting to metastases [31,96], improving therapeutic outcomes. However, although using activated neutrophil shells without therapeutic cargo can neutralize the cytokines that recruit neutrophils to tumors and thereby inhibit tumor metastasis, such particles do not exert therapeutic effects on primary tumors [97]. One possible explanation is that native neutrophils may inadvertently promote tumor proliferation and progression, particularly in gliomas [97,98]. Therefore, it is important to be cautious when using unmodified neutrophil-based delivery systems during cancer treatment. By contrast, neutrophile membrane-coated NPs (NM-coated NPs) show preferential accumulation in arthritic joints compared with other coated or uncoated nanoparticles. This is attributed to the chemotactic properties and adhesion molecule expression of neutrophil membranes, which are naturally home to inflamed tissues via interactions with activated endothelium and inflamed synovium. In a collagen-induced arthritis mouse model, NM-coated NPs exhibited significantly enhanced retention at the inflammation site and superior uptake by chondrocytes compared with RBC-coated controls. Notably, while RBC-coated nanoparticles failed to reduce cartilage erosion or inflammatory cytokine levels, neutrophil-coated BMCNPs showed therapeutic efficacy comparable to conventional treatments, such as anti-TNF-α and anti-IL-1β antibodies, effectively preserving cartilage content and attenuating systemic inflammation [99]. Furthermore, in a more recent study, Duan et al. designed NM-coated NPs loaded with baicalin for the treatment of lipopolysaccharide (LPS)-induced lung inflammation. These NM-coated NPs exhibited enhanced homing to inflamed lung tissues due to natural chemotactic cues and membrane-anchored adhesion molecules, resulting in significant reduction in inflammatory markers, such as IL-6 and TNF-α, in both lung tissues and serum [100].

Regarding platelets, platelet camouflage is widely explored [101,102,103,104,105,106] for tumor-directed delivery. For example, platelet-coated nanoparticles (PM-coated NPs) produced a statistically significant increase in tumor accumulation compared with uncoated NPs and free drug. Moreover, they reduced TNF-α levels to 7.2-fold lower than the free drug, 3.6-fold lower than uncoated NPs, and 2.3-fold lower than the commercial product Taxol^®^, which correlated with reductions in tumor size and weight [107]. Supporting this targeted nature, the tissue distribution of CD44 expression across major organs (heart, liver, spleen, lung, and kidney) and tumor matched the accumulation pattern of PM-coated NPs—with the notable exception of the liver [105]. Thus, nanoparticles with platelet membranes accumulate in tumors via both passive and active targeting. Beyond oncology, inflammation can be caused by ischemia [108]. Accordingly, platelet-camouflaged nanosystems show strong targeting ability to myocardial ischemia–reperfusion injury [109,110]. Consistently, stronger affinity of PM-coated NPs for thrombus sites compared with uncoated NPs has also been demonstrated [111]. For instance, Jin et al. developed PM-coated NPs for inhalation therapy of acute lung injury. These particles not only accumulated and were retained to a greater extent in inflamed lungs, but they also significantly reduced pro-inflammatory cytokine levels and neutrophil infiltration compared with uncoated NPs [112]. In another study, PM-coated NPs showed much higher accumulation than the pure drug in lungs infected with methicillin-resistant Staphylococcus aureus [113].

Shifting focus to homotypic targeting, another goal of targeting is the delivery of drugs to source cells in organisms. Cancer cells possess a mechanism of cell-specific homing preference, which is mostly achieved through adhesion mechanisms [114]. Consistent with this, downregulating levels of integrin αvβ3 significantly decrease uptake by CM-coated NPs [115]. In line with adhesion biology, the high targeting ability of CM-coated NPs toward their source cancer line or culture is attributed to CD44, cadherin-2, and zyxin membrane proteins [116,117]. Consequently, NPs coated with cancer cell membranes inherit these homotypic adhesion capabilities, which allow them to selectively accumulate in tumors derived from the same cell line while displaying minimal affinity for unrelated tumor types [118]. To this end, coating nanoparticles with cancer cell membranes has emerged as a promising strategy to improve homotypic targeting [119]. For example, Lu et al. showed that NPs coated with membranes from U251 glioma cells displayed increased accumulation in orthotopic U251 tumors. Moreover, in a patient-derived xenograft model, tumor targeting was also enhanced when membranes isolated from the xenograft tissue itself were used as the coating source [117].

Moreover, it is important not to overlook the physicochemical characteristics that can influence uptake, particularly by cancer cells. The stiffness of BMCNPs directly affects the mechanism of uptake by cancer cells. Among three RC-NPs with Young’s moduli of 11 MPa, 95 MPa, and 173 MPa, nanoparticles with 95 MPa demonstrated the most effective penetration into cancer cells in vitro and the highest accumulation in tumors in vivo. For stiffer nanoparticles, the mechanism of receptor-mediated endocytosis was predominant, whereas softer nanoparticles could enter cells through both endocytosis and fusion [33]. This dual mechanism of penetration in softer nanoparticles suggests a more versatile uptake pathway compared to their stiffer counterparts.

### 5.2. Targeting via Exogenous Exposure to BMCNPs

#### 5.2.1. Chemical and Physical Modifications

At the same time, differences between healthy and pathological tissues include many specific targets beyond those present on source cell membranes for BMCNPs [120]. Accordingly, the use of small molecules that can easily bind via physical or chemical modification to the nanoparticle surface is an attractive strategy and allows further improvement of targeting. This is especially relevant for RBC-coated NPs, which do not have specific inflammation-targeting molecules on their surface. For instance, the use of a target for (EpCAM)-positive MCF-7 breast cancer cells demonstrated that RBC-coated NPs functionalized by lipid insertion method had a 48-fold higher binding capability compared to non-targeted RBC-coated NPs in vitro [121]. Furthermore, functionalization of RBC-coated NPs by preincubate with cRGD-PEG-DSPE—ligand Integrin αvβ3—enhanced tumor delivery efficiency by 1.9-fold compared with uncoated nanoparticles, whereas RBC-coated NPs without a targeting ligand improved in vivo delivery efficiency by 1.5-fold. Importantly, tumor metabolism activity during treatment decreased by approximately 30% more with the use of the targeted system [122]. In another study, the application of folic acid as a tumor-targeting ligand proved to be 23% more effective in therapy compared with non-targeted particles [123].

Additionally, targeting efficiency can be increased using other types of BMCNPs. In particular, tumor necrosis factor-related apoptosis-inducing ligand (TRAIL) binds to death receptors 4 and 5, which are overexpressed on the surface of various tumor cells, thereby triggering caspases or mitochondrial-dependent cell death without in vivo toxicity. Remarkably, the mean fluorescence intensity of isolated tumor tissues in the TRAIL -NM-coated NPs group was almost twice as high as in the NM-coated NPs group. Moreover, mice in the TRAIL-NM-coated NPs group exhibited the slowest tumor growth during the study period. On the other hand, the NM-coated NPs and the mix of TRAIL with NM-coated NPs groups did not show differences in the tumor growth rate, thus revealing no synergistic effect without drug loading onto the delivery system [124]. Finally, modification of NM-coated NPs with an ApoA-I mimetic peptide increased targeting to the region of rheumatoid arthritis, showing specific affinity for macrophages to deliver a reprogramming agent. Indeed, the use of targeted NPs suppressed synovial inflammation and reduced joint damage in vivo compared with pure substances and non-targeted particles [125].

#### 5.2.2. Genetic Modifications

There is a limit to what can be achieved by chemical modifications. For this reason, it is often much easier to obtain a highly specific receptor on the cell surface using genetic modifications compared to the sequential steps of producing, isolating, and subsequently conjugating recombinant proteins to particles. Moreover, targeting specificity can be further enhanced by engineering donor cells to express desired membrane proteins prior to BMCNP fabrication. For example, preliminary overexpression of the chemokine receptor CXCR4 in stem cells resulted in coating-derived NPs with significantly higher accumulation (~2-fold) in ischemic tissues compared to NPs coated with unmodified stem cell membranes [49]. Other genetic platforms, such as CD63 and its truncated variants [126], PTGFRN [127,128], lactadherin C1C2, Lamp2b, etc. [129], can be used for exhibiting targeting peptides on the surface of biomimetic NPs. For instance, enhanced C-C chemokine receptor type 2 expression at macrophage cells facilitated the chemotaxis of nanoparticles toward inflammatory targets and promoted the healing of spinal cord injuries. In particular, modified NPs demonstrated increased tropism to the spinal cord injury on days 1 and 3, compared with unmodified MM-coated NPs [130]. Moreover, the use of modified macrophage cell membranes with integrin α4/β1 for targeting atherosclerotic plaque and CD47 for additional avoidance of MPS uptake improved delivery efficiency in vivo and reduced phagocytosis in vitro [58]. In addition, the combination of chimeric antigen receptor (CAR) engineering and highly motile neutrophils can preserve their antitumor N1 phenotype and provide excellent therapeutic efficacy in the treatment of glioblastoma (GBM). Specifically, the use of CAR-NM-coated NPs increased drug delivery from 1% for uncoated particles up to 20% [131]. In fact, by day 60 of therapy, survival was 20% in the uncoated NPs and CAR-neutrophil groups, whereas it reached 80% in the CAR-NM-coated NPs group [131]. Finally, it is noteworthy that M2pep is a peptide demonstrating high affinity for M2 macrophages. Notably, M2pep–CM-coated NPs showed superior cargo retention compared with CM-coated NPs, 24 h after injection in vivo, as confirmed by subsequent confocal microscopy data. Consequently, more efficient delivery of the reprogramming agent resulted in a reduced population of M2 macrophages (M2pep–CM-coated NPs 16.6 ± 4.1% vs. 24.7 ± 2.3% CM-coated NPs) [132].

#### 5.2.3. Pre-Activation of Source Cells

Beyond native, chemical, and genetic approaches for obtaining specific membrane-target molecules, pre-activation of the source cell is another effective method. This strategy involves exposing donor cells to defined stimuli, such as pathogens, cytokines, or hypoxic conditions, prior to membrane harvesting. As a result, the cells upregulate specific surface receptors and adhesion molecules that are then inherited by the BMCNPs. For example, pretreated macrophage by *Staphylococcus aureus* or *Escherichia coli* before being used to camouflage NPs increased expression on the surface in vitro of toll-like receptors (TLR) 4 and 2, or only 4, respectively. In addition, subcutaneous administration into the local infection region of Staphylococcus aureus, fluorescently labeled particles showed a faster decay of the fluorescence signal of uncoated NPs (34.89% of the fluorescence intensity remained) when compared to untreated MM-coated NPs (41.37%), Escherichia coli-pretreated MM-coated NPs (48.87%), and Staphylococcus aureus-pretreated MM-coated NPs (65.48%) [133]. Along the same lines, Cao et al. proposed nonviral gene vectors for the treatment of multidrug-resistant bacterial sepsis based on bone marrow macrophage-coated NPs. After treatment of mice with macrophage colony-stimulating factor, macrophages were isolated from the bone marrow. As a result, bone marrow-derived MM–coated NPs rescued 90% of the immunosuppressed mice with sepsis, whereas MM-coated NPs rescued only 66.6%. By comparison, the survival rates of the uncoated and MM-coated NPs-treated groups on day 30 were 65% and 77%, respectively [134]. Finally, a comparison of antimetastatic activity between “activated neutrophil membranes by LPS”-coated NPs and nonactivated NM-coated NPs showed greater efficacy for activated NPs compared to nonactivated [97].

### 5.3. Penetration of Biological Barriers

#### 5.3.1. Endolysosomal Compartment

A major biological barrier in nanomedicine is the endolysosomal compartment, which entraps NPs in endosomes that mature into lysosomes. This process increases intravesicular acidity and activates proteolytic enzymes, leading to NP degradation and cargo loss, significantly limiting their therapeutic efficacy. Conventional strategies to bypass endolysosomal entrapment include the following [135]:

- Proton sponge effect (e.g., PAMAM, PEG-PCL-PEI, PPTS);
- Osmotic lysis (e.g., PMPC-b-PDPAEMA, DOPA, DOTAP, CaP);
- Swelling-induced escape (e.g., PDEAEMA, PAEMA, PEGDMA, mPEG);
- Pore formation (e.g., LPEI polyplexes, PEI, gp41 peptide);
- Membrane disruption (e.g., DSPE-PCB, ultrasound-responsive polymersomes, temperature-sensitive bubble liposomes);
- Membrane fusion and photochemical internalization.

Alternatively, lysosomal avoidance can be achieved by redirecting NPs toward cavosome-mediated transport or Golgi/ER trafficking, as demonstrated with histone/PEI polyplexes, transferrin-PEI, KDEL-modified gold NPs, and ER membrane-decorated liposomes.

BMCNPs exhibit unique endolysosomal escape behaviors. While membrane-coated NPs are initially entrapped in lysosomes, they show a distinct ability to exit and deliver cargo. Cationic lipid modifications enhance lysosomal entrapment but also promote subsequent escape [128,136]. In contrast, unmodified biomimetic NPs exhibit minimal lysosomal colocalization, suggesting direct membrane fusion with target cells and cytoplasmic cargo release. Supporting this, cathepsin inhibition—which boosts hybrid NP delivery—has no effect on unmodified biomimetic NPs, indicating divergent internalization pathways [128].

These findings highlight the differential impact of the endolysosomal barrier on BMCNPs, depending on their composition, and underscore the need for tailored engineering strategies to optimize intracellular delivery.

#### 5.3.2. Blood–Brain Barrier

One of the most significant challenges in targeted drug delivery is transporting therapeutic agents across the blood–brain barrier (BBB), a tightly regulated and selective interface that separates the circulating blood from the brain’s extracellular environment. The BBB is composed primarily of endothelial cells connected by tight junctions, supported by pericytes and astrocytic end-feet, forming a physical and biochemical shield that protects CNS while maintaining homeostasis [137]. However, this protective role presents a major obstacle for drug delivery, as most therapeutic agents—particularly large or hydrophilic molecules—are unable to cross the BBB in sufficient concentrations to exert pharmacological effects [138]. Passive diffusion is limited to small, lipophilic molecules, and even these are often subject to active efflux by transport proteins such as P-glycoprotein [139].

BMCNPs offer a promising strategy for safely and effectively crossing the BBB. By utilizing cell types that naturally traffic across the BBB, such as RBCs, immune cells (e.g., macrophages or neutrophils), stem cells, tumor cells, or microglia, or by engineering synthetic targeting ligands, BMCNPs can achieve improved BBB permeability and brain-specific accumulation [140]. The ability of BMCNPs to penetrate the BBB is determined largely by the origin of the coating membrane and the membrane-associated surface proteins. For example, incorporation of targeting peptides, such as apolipoprotein E (ApoE), which binds low-density lipoprotein receptors (LDLRs) expressed on both BBB endothelial cells and glioblastoma cells [141], has shown high potential for enhancing brain delivery [142]. PEGylated ApoE-functionalized BMCNPs demonstrated significantly extended circulation times in glioblastoma-bearing animals (8.2 h vs. 6.7 h) compared to non-targeted BMCNPs [143]. In contrast, non-PEGylated ApoE peptide did not alter pharmacokinetic profiles compared to untargeted particles [144], reinforcing the importance of PEG in extending systemic circulation [145]. A similar effect was observed with Angiopep-2 (Ang) and lexiscan (Lex), an adenosine receptor agonist recently shown to transiently open the BBB. PEGylated Ang-RBC-Lex NPs showed increased half-life (t_1_/_2_ = 9.3 h) compared to non-PEGylated RBC-Lex NPs (t_1_/_2_ = 7.8 h) [146]. Notably, in all studies mentioned above, BMCNP accumulation was significantly higher in tumor-bearing brain tissue compared to healthy brain, confirming the importance of pathological cues in enhancing targeting efficacy.

It is important to note that the choice of targeting molecules can substantially influence the biodistribution of NPs. For instance, ischemia–reperfusion injury is known to induce overexpression of stromal cell-derived factor-1 (SDF-1) in the affected regions, which activates the CXCR4/SDF-1 chemokine axis. This signaling pathway promotes the homing and accumulation of CXCR4-expressing endothelial cells at the site of injury [147]. Building on this mechanism, coating nanoparticles with membranes that overexpress the CXCR4 receptor has been proposed as a strategy to enhance targeting toward ischemic tissues [148]. Remarkably, this approach resulted in a threefold increase in nanoparticle accumulation within ischemic areas compared to nanoparticles lacking CXCR4 expression. However, the authors also observed elevated fluorescence signals in the lungs, kidneys, and liver of healthy animals treated with CXCR4-functionalized nanoparticles. This unexpected biodistribution was hypothesized to be related to increased SDF-1 expression in these organs, potentially as a systemic response to ischemic injury. These findings highlight both the targeting potential and off-target considerations associated with chemokine-receptor-based delivery strategies.

Under pathological conditions, such as GBM or neuroinflammation, the integrity of the BBB is compromised, facilitating the infiltration of circulating immune cells, particularly monocytes and macrophages (Table 3). A key mechanism enabling this process involves the upregulation of endothelial adhesion molecules (e.g., ICAM-1), which engage integrins on immune cells to mediate a multi-step adhesion and transmigration cascade. Among the most relevant integrins are Integrin α4 and Mac-1, which bind to ICAMs on activated endothelium and support rolling, firm adhesion, and diapedesis across the BBB [149]. The glioma microenvironment further enhances this process by secreting high levels of chemokines and growth factors, such as CCL2, CSF-1, and VEGF, which not only recruit circulating monocytes but also increase BBB permeability [150]. Inflammatory diseases, such as multiple sclerosis and viral encephalitis, similarly promote integrin-dependent macrophage recruitment to the CNS [151].

Leveraging this natural trafficking pathway, macrophage BMCNPs have emerged as a promising strategy for targeted drug delivery to GBM [46,47,152]. These BMCNPs not only exhibit enhanced BBB penetration but also demonstrate prolonged circulation and improved pharmacokinetics. For example, macrophage-coated NPs showed significantly higher blood retention (12.3% vs. 6.9% at 24 h) and extended half-life (3.37 h vs. 1.52 h) compared to uncoated controls, along with reduced inflammatory marker expression, indicating improved biocompatibility. In this study, the organ distribution profile for coated and uncoated nanoparticles was quite similar, with the liver and spleen the main sites of BMCNP accumulation, although the contents of the target substance in these organs were lower than for control particles [152]. Interestingly, the incorporation of targeting peptides such as Ang into BMCNP design significantly reduces off-target accumulation in the liver, spleen, lungs, and kidneys, while simultaneously enhancing brain-specific delivery [46].

## 6. Therapeutic Efficiency

As previously noted, membrane coating significantly enhances the performance of NPs as drug delivery platforms by improving their circulation time, immune evasion, and tissue targeting. However, beyond these delivery-related benefits, BMCNPs can also intrinsically contribute to therapeutic efficacy. This enhancement occurs not only through improved targeting but also via the protective properties conferred by the membrane itself, which can stabilize and preserve the functional integrity of the nanoparticle core.

For instance, certain inorganic nanomaterials, such as tungsten oxide (W_18_O_49_), are known to rapidly oxidize in physiological environments, leading to the release of various ions that compromise their effectiveness as photodynamic therapy (PDT) agents. To overcome this, platelet membrane coating was applied to protect the W_18_O_49_ core from oxidative degradation, thereby preserving its therapeutic potential [153].

Furthermore, the membranes themselves may impart unique biological functionalities to the BMCNPs. These can include immunomodulatory, targeting, or adhesive properties, depending on the cellular source of the membrane, thereby expanding the therapeutic utility of the platform beyond its role as a passive carrier.

Cancer immunotherapy is a treatment strategy that harnesses the body’s immune system to recognize and eliminate tumor cells [154,155]. Cancer vaccines, a specific subset of immunotherapy, are designed to elicit or enhance immune responses against tumor-specific or tumor-associated antigens [156].

Despite encouraging progress, the clinical efficacy of cancer vaccines remains limited by several barriers, including poor delivery efficiency, immunosuppressive tumor microenvironments, and challenges in selecting optimal antigens. These limitations highlight the need for advanced delivery platforms, such as nanoparticle-based systems, to enhance antigen presentation and immune activation [157,158]. As previously noted, many synthetic nanomaterials exhibit the ABC phenomenon upon repeated administration. Given that immunotherapy protocols often require multiple dosing cycles, it would be prudent to implement BMCNPs in such applications, as they offer improved biocompatibility and circulation profiles over conventional formulations.

The initiation of an effective anti-tumor immune response in cancer immuno-therapy often relies on the activation and maturation of dendritic cells (DCs). Upon uptake of Tumor-Associated Antigens (TAAs) released by dying tumor cells or delivered via vaccines or nanocarriers, immature DCs undergo maturation, upregulating major histocompatibility complex (MHC) molecules, co-stimulatory markers (e.g., CD80, CD86), and proinflammatory cytokines. Mature DCs then migrate to the draining lymph nodes, where they present processed tumor peptides via MHC class I and II molecules to naïve CD8^+^ and CD4^+^ T cells, respectively. This process, combined with co-stimulatory signaling and cytokine secretion (e.g., IL-12), promotes the clonal expansion and differentiation of cytotoxic T lymphocytes (CTLs) and helper T cells. Activated CTLs subsequently traffic to the tumor site, where they recognize and kill tumor cells expressing cognate antigens [159].

One strategy in cancer immunotherapy involves the activation of DCs within lymph nodes, often achieved through the use of nanoparticle-based delivery systems. When designing such platforms, particle size plays a critical role, as the optimal range for lymph node targeting and efficient uptake by node-resident DCs is approximately 10–100 nm [160]. In this context, Gan et al. developed a delivery system based on melanoma B16F10 cancer cell membrane-coated nanoparticles loaded with CpG, an immunomodulator and TLR9 agonist [160]. While both BMCNPs and vesicles lacking an inorganic core exhibited similar lymph node accumulation, the BMCNP-treated group demonstrated significantly superior immunotherapeutic efficacy in vivo.

Specifically, the number of tumor antigen- and CpG-double-positive macrophages and DCs was statistically higher in this group following a single administration, suggesting not only successful co-delivery of CpG and tumor antigen, but also enhanced immune cell activation mediated by interactions with the tumor cell membrane. Furthermore, even in the absence of immunostimulatory drugs, tumor cell-derived BMCNPs alone were shown to promote DC maturation and antigen presentation, exhibiting intrinsic antitumor vaccine-like properties [161].

Additional improvements in DC activation can be achieved using hybrid membrane coatings that combine bacterial membranes, which are rich in pathogen-associated molecular patterns (PAMPs), with tumor cell membranes. This approach leverages interactions between bacterial PAMPs and DC pattern recognition receptors to promote phagocytosis and immune activation [162,163]. Together, these studies highlight the potential of BMCNPs not only as passive delivery vehicles but also as active immunotherapeutic agents that can drive DC activation and enhance antitumor immune responses. In addition, researchers have proposed a biomimetic nanoscale artificial APC platform based on the wild-type B16-F10 murine melanoma cell line, which is known to naturally express MHC class I molecules. To enhance its immunostimulatory capacity, the cells were genetically engineered to co-express CD80 and a TAA on the membrane surface, thereby mimicking the functional properties of DCs to promote multispecific T-cell activation. Subcutaneous pre-injection of such BMCNPs was shown to effectively induce tumor antigen-specific immune responses in vivo [164].

Another promising strategy in cancer immunotherapy involves the use of pre-activated DCs as a source of membrane coating. Cheng et al. [165] developed a nanoparticle system coated with vesicles derived from DCs that had been pre-matured in vitro using tumor lysate. This pre-activation strategy allowed the delivery system to retain its immunostimulatory capacity without significantly increasing particle size. Importantly, the use of these DC membrane-coated nanoparticles successfully inhibited tumor growth and prevented metastasis in an ovarian cancer model.

The tumor microenvironment (TME) is a complex ecosystem composed of cancer cells, stromal cells, immune cells, extracellular matrix, and signaling molecules that collectively influence tumor progression and therapeutic response. A hallmark of the TME is its immunosuppressive nature, which facilitates tumor immune evasion by inhibiting cytotoxic T cell activity, promoting regulatory T cells, and recruiting immunosuppressive myeloid populations, such as tumor-associated macrophages (TAMs) [166,167]. TAMs are among the most abundant immune cells in the TME and exhibit functional plasticity. They are broadly categorized into two phenotypes: M1-like TAMs, which are pro-inflammatory and exert antitumor effects, and M2-like TAMs, which promote tumor growth, angiogenesis, and immunosuppression. High infiltration of M2-polarized TAMs is often associated with poor prognosis in various cancers [168].

The immunomodulatory potential of BMCNPs has been demonstrated in several studies involving macrophage polarization and inflammatory disease models. For example, PLGA nanoparticles coated with membranes from pure pre-activated macrophages were shown to modulate macrophage polarization within tumor tissues. Specifically, the percentage of M1 macrophages increased from 11.27% to 13.34%, while M2 macrophages decreased from 31.10% to 29.14%, resulting in an elevated M1/M2 ratio, which is indicative of a shift toward a pro-inflammatory, anti-tumor microenvironment [169].

Further evidence supports the use of natural killer cell membranes (NKCMs) to reprogram macrophage phenotypes. Proteomic analysis has revealed that NKCMs contain proteins such as IRGM1, CB1, Galectin-12, RAB-10, and RANKL, which are capable of engaging macrophage surface receptors (e.g., TLR 4 or tumor necrosis factor receptor) to induce or enhance M1-type polarization. Accordingly, NPs coated with NKCMs significantly upregulated M1 markers (iNOS/CD86) and downregulated the M2 marker CD206 in vitro compared to uncoated controls [170]. This shift in macrophage phenotype was accompanied in vivo by enhanced DC maturation, an increase in helper T-cell populations, and greater T-cell infiltration into tumor tissue—hallmarks of effective cancer immunotherapy.

Conversely, in diseases characterized by chronic inflammation, such as osteoarthritis, the therapeutic goal may be to suppress pro-inflammatory signaling. In these cases, reversing macrophage polarization from an M1- to an M2-dominant phenotype is considered beneficial. For instance, a reduced M1/M2 ratio was associated with a significant decrease in synovial inflammation and cartilage matrix degradation in a murine model of osteoarthritis [171]. In a related study, NPs coated with membranes derived from pre-polarized M2-type macrophages increased the presence of CD206^+^ (M2) macrophages and reduced CD86^+^ (M1) macrophages at the site of inflammation. This shift correlated with lower synovitis scores and greater therapeutic efficacy compared to uncoated particles, despite both formulations containing the same reprogramming agent [172].

Regarding liver disease, an increase in the M2 macrophage subpopulation—from 0.26% to 13.5%—was observed in the group of animals with acute liver failure that received M2-coated NPs. This result indicates that the enhanced efficacy of BMCNP-based therapy stems not only from its targeting capability but also from the intrinsic properties of the cell membrane [173].

Interestingly, in another study, BMCNPs without any therapeutic payload were shown to exhibit inherent efficacy in the treatment of arthritis [99]. NM-coated NPs, when compared to RBC-coated controls, demonstrated superior accumulation in inflamed joints, enhanced uptake by chondrocytes in vivo, and reduced cartilage damage on histological slices in vitro. While RBC-coated nanoparticles showed minimal therapeutic impact, neutrophil-BMCNPs performed comparably to positive controls, such as anti-IL-1β and anti-TNF-α therapies, in preserving cartilage content and attenuating systemic inflammatory responses. These findings underscore the potential of membrane selection in optimizing the therapeutic efficacy of BMCNPs for inflammatory diseases.

Notably, an intriguing application of RBC-coated NPs was proposed by Hu et al. Specifically, crosslinking RBC-coated NPs with valve tissue not only preserved the advantages of glutaraldehyde-based coating (namely, structural stability and mechanical properties) but also demonstrated enhanced functionalities, including anti-coagulation, anti-inflammation, anti-calcification, and endothelialization [174]. Furthermore, Su et al. revealed that PM-coated NPs alone were able to reduce mitotic activity in cardiomyocytes within injured hearts after 4 weeks; these cells were also undergoing cytokinesis. Treatment with platelet-based nanoparticles decreased scar size and exerted a statistically significant impact on therapy outcomes. Importantly, the findings suggest that the therapeutic effect of PM-coated NPs may be attributed to the activation of Nkx2.5-positive cells and endothelial progenitor cells, as well as to the stimulation of neovascularization [175].

The application of stem cell membrane-coated nanoparticles has shown promising results in regenerative medicine, particularly in wound healing. In one study, the use of stem cell membrane-coated PLGA-PEI nanoparticles for miRNA delivery significantly enhanced therapeutic outcomes compared to other formulations. The group treated with Stem-Cell-Membrane-Coated PLGA-PEI-miRNA NPs exhibited the highest rate of wound closure, the narrowest epithelial gap, the greatest number of mature blood vessels, and the most newly formed hair follicles and sebaceous glands. These outcomes surpassed those achieved by saline, free miRNA, or stem cell vesicle–miRNA formulations, suggesting that the Stem-Cell-Membrane-Coated PLGA-PEI system offers the most effective strategy for promoting tissue regeneration [176]. To further understand the advantages of incorporating an inorganic polymeric core, a comparison was made between nanoparticles with PLGA-PEI and without the PLGA-PEI core. Both systems demonstrated comparable physicochemical properties, including stability in 10% fetal bovine serum. Their hydrodynamic diameters measured approximately 198.4 nm and 172.4 nm, respectively, and their zeta potentials were −10 mV and −19 mV. However, the PLGA-PEI-based nanoparticles exhibited a notably higher miRNA encapsulation efficiency (approximately 90% vs. 60%), which may be attributed to differences in surface charge. The less negative zeta potential of PLGA-PEI NPs may facilitate improved electrostatic interactions with the negatively charged miRNA, thereby enhancing loading efficiency.

## 7. Conclusions

BMCNPs represent a biohybrid system that combines a synthetic nanoparticle core with a natural cell membrane, thereby uniting the engineering versatility of nanomaterials with the biological functions of living cells. Such a design provides prolonged circulation and immune evasion, primarily through the presence of CD47 and other “self” signals, which collectively extend the half-life of nanoparticles several-fold. At the same time, BMCNPs exhibit excellent biocompatibility, a factor that is critically important for the clinical translation of nanomedicines.

Beyond improving circulation, membrane coating also imparts biological functionality. The membrane layer may carry proteins and receptors capable of conferring tissue-specific targeting or immunomodulation. Importantly, the selection of the source cell enables precise tissue homing and the ability to traverse biological barriers, such as the BBB. For example, macrophage-derived coatings enhance accumulation in infectious foci, neutrophil membranes facilitate targeting to sites of systemic inflammation, whereas cancer cell membranes provide homotypic tropism. Moreover, additional modification of membranes, including genetic engineering, offers opportunities to further enhance the targeting specificity and functional properties of these nanocarriers.

Nevertheless, the fabrication of BMCNPs poses significant challenges. Fusion of membranes with nanoparticle cores must occur under carefully controlled conditions, yet in practice, complete coating is achieved for only a small fraction of particles (1.8–6.5%). Incomplete coverage inevitably increases macrophage uptake and shortens nanoparticle half-life. Other hurdles include maintaining sterility, preserving the functional integrity of membrane proteins, ensuring storage stability, and developing standardized production protocols that would guarantee reproducibility across batches [177,178].

It should be emphasized, however, that the short lifespan, high probability of apoptosis ex vivo, and resistance to genome editing create significant challenges for developing such delivery systems [179]. Therefore, although CAR-NM-NPs significantly slowed tumor growth in mice, the difference in survival between experimental groups compared with CAR-neutrophils and uncoated NPs remained minimal. Importantly, reducing the time of cell isolation and preparation for injection to 1 h increased survival in the CAR-neutrophil group, while dose escalation and multiple injections led to higher survival in the CAR-NM-NP group.

Undoubtedly, recent advances in BMCNP research raise hopes for the successful clinical translation of coated nanoparticles in the future. However, several safety and standardization challenges must be addressed before this can be achieved.

First, the manufacturing technology for BMCNPs is not yet fully developed for large-scale production, which hinders their translation to clinical studies. Changes in fabrication methods can lead, for example, to differences in the integrity of membrane coating, leading to unpredictable changes in performance after distribution into the body.

Second, characterization methods for BMCNPs are limited. Cell characteristics can vary at different growth stages, altering the properties of cell membranes and membrane proteins and leading to batch-to-batch variability.

Third, the use of BMCNPs carries inherent risks connected to the presence of minor protein or DNA components, leading to undesired side effects on patient cells, including uncontrolled proliferation and differentiation, cellular embolism, and autoimmune reactions.

Fourth, the properties of proteins may change during long-term storage, which is a key factor in BMCNP efficacy. Therefore, developing standardized storage protocols also remains a critical task for future research.

## Figures and Tables

**Table 1:** The t_1/2_ prolongation of nanomaterials after cell coating.

Source Cells for Membrane Coating	Core	t_1/2_ (Elimination Phase) Before Coating vs. After Coating, h	Animal Model	Route of Injection	Total Clearance Cl	Volume of Distribution Vd	References
RBCs	Gold nanorods (L~50 nm)	0.980 vs. 14.9	Male BALB/c mice	Intravenous injection	0.641 vs. 0.024 L/h	0.898 vs. 0.511 L	[7]
	PLGA NPs (d = 240 nm)	0.41 vs. 1.08	C57BL/6J mice	Retroorbital injection	NS	NS	[21]
	Perfluorocarbon encapsulated within PLGA NPs (d~400 nm)	~7 vs. 13.93	Female nude mice	Subcutaneous injection	NS	NS	[22]
	PLGA NPs (d = 80 nm)	15.8 vs. 39.6	Male ICR mice	Intravenous injection	NS	NS	[23]
	Magnetic NPs (d = 52 nm)	2.6 vs. 8.1	Female C57BL/6 mice with ID8 tumor	Intravenous injection	NS	NS	[24]
	PLGA NPs (d = 120 nm)	28.2 vs. 51.1	BALB/c mice	Intravenous injection	NS	NS	[25]
	Melanin NPs (d = 216 nm)	4.0 vs. 11.2	Female ICR mice	Intravenous injection	NS	NS	[26]
	Lipid multichambered NPs (d = 220 nm)	~10 vs. ~25	Tumor-bearing nude mice	Intravenous injection	~2 vs. <1 L/h/kg	NS	[27]
	Au nanocages (d~150 nm)	~8.2 vs. 29.528	Female BALB/c mice with tumor	Subcutaneous injection	0.021 (nmol)/(nmol/mL)·h	0.883 (nmol)/(n mol/mL)	[28]
Macrophages	Amphiphilic oxidation-sensitive chitosan oligosaccharide NPs (d = 149 nm)	5.43 vs. 9.82	ApoE−/− mice	Intravenous injection	NS	NS	[29]
	Baicalin liposomes (d = 182 nm)	4 vs. 4.7	Rats	Intravenous injection	0.0070 vs. 0.0032 L/min/kg	2.29 vs. 0.62 L/kg	[30]
Neutrophiles	PLGA NPs (d~75 nm)	0.77 vs. 6.59	Male SD rats	Intravenous injection	3160.37 vs. 17.42 mL/h/kg	3397.85 vs. 144.2	[31]
Cancer cells	Magnetic NPs (d = 52 nm)	2.6 vs. 4.4	Female C57BL/6 mice with ID8 tumor	Intravenous injection	NS	NS	[24]
	SiO2 NPs (d = 120 nm)	9.1 vs. 23.6	SD rats	Intravenous injection	0.062 vs. 0.07 L/kg	0.017 vs. 0.004 L/h/kg	[10]
	Melanin NPs (d = 216 nm)	4.0 vs. 5.1	Female ICR mice	Intravenous injection	NS	NS	[26]
RBCs + cancer cells	Magnetic NPs (d = 52 nm)	2.6 vs. 7.1	Female C57BL/6 mice with ID8 tumor	Intravenous injection	NS	NS	[24]
RBCs + cancer cells (2:1)	Melanin NPs (d = 216 nm)	4.0 vs. 10.9	Female ICR mice	Intravenous injection	NS	NS	[26]
RBCs + cancer cells (1:1)	Melanin NPs (d = 216 nm)	4.0 vs. 10.7	Female ICR mice	Intravenous injection	NS	NS	[26]

**Table 2:** QTPP of BMCNPs.

CMAs	Target	Justification
Size	<100 nm	Optimal size for reducing non-target organ accumulation [74], maximize t_1/2_, and reduce uptake by MPS
Coating integrity	100%	Maximize t_1/2_, reduce uptake by MPS, and enhance tumor accumulation [10]
CD47	Presence	Maximize t_1/2_, reduce uptake by MPS
Stiffness	~10 MPa	Maximize t_1/2_, reduce uptake by MPS, and enhance tumor accumulation [25]

**Table 3:** Crossing BBB strategies of BMCNPs.

Pathological Conditions	Membrane Source	BBB Penetration Mechanism	Main Effect	References
Ischemia–reperfusion injury	CXCR4-overexpressing primary mouse thoracic aorta endothelial cells	CXCR4/SDF-1 chemokine axis	~3-fold increase in accumulation at ischemic region vs. control NPs	[147]
Glioma	RBCs	ApoE–LDLR- mediated uptake	Tumor accumulation of ApoE-BMCNP treatment reached 9.3% of the injected dose per gram of tissue, which was 1.7–2.9-fold higher than those of BMCNPs and uncoated NPs	[143]
Glioma	RBCs	ApoE–LDLR- mediated uptake	ApoE-BMCNP accumulation in the tumor area was enhanced 7-fold and 21-fold compared to BMCNPs and uncoated NPs, respectively	[144]
Glioma	RBCs	Ang-2 and Lex-mediated uptake	Ang-RBC-Lex NPs, which were 2.5-, 2.9–, 3.5-fold higher than that of RBC-Lex NPs, Ang-RBC NPs, and NPs-Lex	[146]
Glioma	Macrophages	Integrin-dependent macrophage penetration	~4-fold increase in accumulation at tumor region vs. uncoated NPs	[47]
Glioma	Macrophages	Integrin-dependent macrophage penetration	At the same time points post-injection, BMCNPs displayed significantly higher accumulation than uncoated NPs	[152]
Glioma	Macrophages	Integrin-dependent macrophage penetration and Ang-2-mediated uptake	The distribution of Ang-BMCNPs in the brain was significantly higher than BMCNPs	[46]

## Abbreviations

The following abbreviations are used in this manuscript:

BMCNPs	Biomimetic cell membrane-coated nanoparticles
NPs	Nanoparticles
PLGA	Poly(lactic-co-glycolic acid)
RBC	Red blood cells
UC	Ultracentrifugation
DGU	Density gradient ultracentrifugation
FNC	Flash NanoComplexation
Half-time	t_1/2_
MM-coated NPs	Macrophage membrane-coated nanoparticles
SM-coated NPs	Stem cell-coated nanoparticles
CM-coated NPs	Cancer cell-coated nanoparticles
CNS	Central nervous system
PS	Phosphatidylserine
ABC	Accelerated blood clearance
ROS	Reactive oxygen species
CMAs	Critical material attributes
QTPP	Quality Target Product Profile
PEG	Polyethylene glycol
EPR	Enhanced permeability and retention
VCAM-1	Vascular cell adhesion molecule 1
ICAM-1	Intercellular adhesion molecule 1
TME	Tumor microenvironment
NM-coated NPs	Neutrophile membrane-coated NPs
LPS	Lipopolysaccharide
PM-coated NPs	Platelet-coated nanoparticles
TRAIL	Tumor necrosis factor-related apoptosis-inducing ligand
CAR	Chimeric antigen receptors
GBM	Glioblastoma
TLR	Toll-like receptors
BBB	Blood–brain barrier
ApoE	Apolipoprotein E
LDLRs	Low-density lipoprotein receptors
Ang	Angiopep-2
Lex	Lexiscan
SDF-1	Stromal cell-derived factor-1
PDT	Photodynamic therapy
DCs	Dendritic cells
TAAs	Tumor-Associated Antigens
MHC	Major histocompatibility complex
CTLs	Cytotoxic T lymphocytes
PAMPs	Pathogen-associated molecular patterns
TAMs	Tumor-associated macrophages
